# Critical assessment of infants born to mothers with drug resistant tuberculosis

**DOI:** 10.1016/j.eclinm.2024.102821

**Published:** 2024-09-05

**Authors:** Marian Loveday, Neel R. Gandhi, Palwasha Y. Khan, Grant Theron, Sindisiwe Hlangu, Kerry Holloway, Sunitha Chotoo, Nalini Singh, Ben J. Marais

**Affiliations:** a HIV and Other Infectious Diseases Research Unit (HIDRU), South African Medical Research Council, Durban, South Africa; b CAPRISA-MRC HIV-TB Pathogenesis and Treatment Research Unit, Durban, South Africa; c Centre for Health Systems Research & Development, University of the Free State, South Africa; d Rollins School of Public Health and Emory School of Medicine, Emory University, Atlanta, USA; e London School of Hygiene and Tropical Medicine, London, UK; f Africa Health Research Institute, Durban, South Africa; g DSI-NRF Centre of Excellence for Biomedical Tuberculosis Research, South African Medical Research Council Centre for Tuberculosis Research, Division of Molecular Biology and Human Genetics, Faculty of Medicine and Health Sciences, Stellenbosch University, Cape Town, South Africa; h King Dinuzulu Hospital Complex, Sydenham, Durban, South Africa; i WHO Collaborating Centre for Tuberculosis, Sydney Infectious Diseases Institute, University of Sydney, Sydney, NSW, Australia; j The Children’s Hospital at Westmead, Sydney, NSW, Australia

**Keywords:** Tuberculosis, TB, Transmission, Mother-to-child, Perinatal, Congenital, Infant, Pregnancy

## Abstract

**Background:**

There have been no detailed descriptions of infants born to mothers treated for drug resistant TB in pregnancy. Critical case history assessment is important to identify risks and guide clinical practice.

**Methods:**

In a cohort of pregnant women with multidrug or rifampicin resistant (MDR/RR)-TB enrolled between 1 January 2013 and 31 December 2022, we followed mother-infant pairs until the infant was 12 months old. We performed critical case history assessments to explore potential mechanisms of *Mycobacterium tuberculosis* transmission to the infant, and to describe the clinical presentation and disease trajectories observed in infants diagnosed with TB.

**Findings:**

Among 101 mother-infant pairs, 23 (23%) included infants diagnosed with TB disease; 16 were clinically diagnosed and seven had microbiological confirmation (five MDR/RR-TB, two drug-susceptible TB). A positive maternal sputum culture at the time of delivery was significantly associated with infant TB risk (p = 0.023). Of the 12 infants diagnosed with TB in the first three months of life, seven (58%) of the mothers were culture positive at delivery; of whom four reported poor TB treatment adherence. However, health system failures, including failing to diagnose and treat maternal MDR/RR-TB, inadequate screening of newborns at birth, not providing appropriate TB preventive therapy (TPT), and *M. tuberculosis* transmission from non-maternal sources also contributed to TB development in infants.

**Interpretation:**

Infants born to mothers with MDR/RR-TB are at greatest risk if maternal adherence to MDR/RR-TB treatment or antiretroviral therapy (ART) is sub-optimal. In a high TB incidence setting, infants are also at risk of non-maternal household and community transmission. Ensuring maternal TB diagnosis and appropriate treatment, together with adequate TB screening and prevention in all babies born to mothers or households with TB will minimise the risk of infant TB disease development.

**Funding:**

10.13039/501100001322South African Medical Research Council.

**Research in context**

**Evidence before this study**

We searched PubMed with the terms “mother-to-child” OR “vertical” OR “congenital” OR “perinatal” AND “transmission” AND “TB” OR “tuberculosis” for articles published in English up to May 1, 2024. Of the 1339 articles retrieved, only 16 were of relevance; 10 were individual case reports or small case series (<5 cases) and the rest were expert reviews. Overall, there is limited evidence to understand risk and guide clinical practice related to perinatal tuberculosis which is exacerbated in pregnant women with MDR/RR TB. A better understanding of transmission dynamics is needed to inform strategies for reducing perinatal transmission and detailed descriptions of mother-infant pairs are important to further our understanding of transmission and disease risk.

**Added value of this study**

For the first time, we demonstrate high rates of infant tuberculosis (TB) disease in prospectively followed mother-infant pairs, where the mothers had microbiologically confirmed MDR/RR-TB. We provide detailed case descriptions, explored the likely mechanisms of transmission, and found that in over half the infants who developed TB in the first three months of life, the mother was culture positive at delivery. Case descriptions suggest that sub-optimal maternal adherence to MDR/RR-TB treatment and antiretroviral therapy (ART) contributed to delayed culture conversion, infectiousness, and transmission. We document how the compromised quality of perinatal health services provided in high TB incidence settings with limited resources contribute to infant TB. Failure to diagnose and adequately treat all mothers with MDR/RR-TB during pregnancy and postpartum, as well as limited newborn screening and provision of appropriate TB Preventive Therapy (TPT) contributed to TB in several infants. Non-maternal household transmission, as well as possible infection in the community, also contributed to infant TB.

**Implications of all the available evidence**

In high TB incidence settings, there should be a low threshold for screening pregnant women for TB, with the inclusion of a World Health Organization (WHO) approved nucleic acid amplification test (NAAT) to detect both TB and MDR/RR-TB. All babies born to mothers with TB should be screened for disease in a systematic fashion and considered for appropriate TPT as recommended by the WHO. Close contacts of the mother-infant pair should also be screened for TB and considered as potential alternative sources of TB infection.

It is important to ensure maternal adherence to TB treatment and ART during pregnancy, delivery and the postpartum period, which requires additional counselling and adherence support to assist women and protect their babies during this difficult period. More research and better evidence to improve the management of pregnant women with TB and in particular infants born to mothers with MDR/RR-TB are urgently needed.

## Introduction

Tuberculosis is a complex disease caused by the airborne transmission of the bacillus *Mycobacterium tuberculosis* complex (*M. tuberculosis*). Progress towards TB control is hampered by our limited understanding *M. tuberculosis* transmission,^1^, ^2^, ^3^ especially in settings with a high burden of HIV and high TB incidence rates, where infection in the community and repeated re-infection is common.^4^ Historically, households were considered the major locus of *M. tuberculosis* transmission.^5^ However, studies from high TB incidence countries in sub-Saharan Africa have demonstrated that transmission mainly resulted from community exposure, where the source of infection was unknown.^6^, ^7^, ^8^, ^9^ including in young children.^10^^,^^11^ When a young child (<5 years old) is identified as a close contact of an infectious TB patient and that child develops TB, the disease usually occurs within 12 months of exposure and is often detected at the time of the initial contact investigation.^12^ In infants with limited social contact, the greatest risk of infection is still considered to be through postnatal transmission from the mother or other caregivers,^13^^,^^14^ while newborn babies may also be infected transplacentally or at birth.

Effective treatment of pregnant women is critical to prevent adverse maternal and neonatal outcomes,^15^, ^16^, ^17^ such as low birth weight, perinatal TB, prolonged hospitalization, and death.^15^^,^^18^ However, optimal management of the mother-infant pair is complex and rarely discussed. This is important given the risk of *M. tuberculosis* transmission, our limited understanding of infectiousness and the likely routes of transmission, as well as the recognised value of breastfeeding for infant survival and maternal infant bonding.^19^^,^^20^ A positive early mother–infant relationship is crucial to optimal child development, emotional security, social competence, autonomy, and intellectual achievement;^21^^,^^22^ thus, a holistic management approach is essential, whilst minimising the risk of *M. tuberculosis* transmission to the infant.

A better understanding of likely transmission dynamics is needed to inform strategies that reduce the infection risk in babies born to mothers with TB. The unique biological link and close social nature of the bonds in the mother-infant pair provides an opportunity to critically explore likely transmission pathways.^23^ An analysis of maternal treatment, as well as pregnancy and infant outcomes, in a cohort of pregnant women with multidrug/rifampicin-resistant TB (MDR/RR-TB) and a high rate of HIV co-infection, demonstrated that more than 20% of infants born to mothers with MDR/RR-TB were diagnosed with TB during the first year of life.^24^ However, in the initial analysis we were unable to interrogate the detailed case histories of all mother-infant pairs, which limited our insight into different infant disease manifestations and relevant risk factors for infant disease.

In this study we performed a critical case history assessment of all mother-infant pairs, where the infant was diagnosed with TB disease, in a cohort of pregnant women diagnosed with MDR/RR-TB. The study aim was to explore possible *M. tuberculosis* transmission routes and to provide detailed descriptions of the infants’ clinical presentation and disease trajectories according to the most likely *M. tuberculosis* transmission route.

## Methods

### Study setting and cohort follow-up

We enrolled pregnant women with MDR/RR-TB diagnosed from 1 January 2013 to 31 December 2022 in the KwaZulu-Natal province of South Africa. All women and their babies were followed through pregnancy, delivery and postpartum until the infant was 12 months old. Detailed methods have been previously published.^25^

### Data capture: mothers

The clinical details captured for each woman included HIV and ART status, resistance pattern and details of their MDR/RR-TB treatment, including their documented treatment response (monthly sputum culture results). The regimens women received, which changed during the study period, are detailed in Supplementary Table S1. The strong relationships developed with mothers during the study period allowed us to have honest discussions about adherence to both MDR/RR-TB treatment and ART, which were captured as self-reported adherence data.

### Data capture: infants

According to country guidelines at the time, all infants born to mothers with TB should have been screened for TB at birth with a chest radiograph and gastric aspirate sent for an Xpert MTB/RIF test (Cepheid, Sunnyvale, CA, USA) and, in the absence of TB disease, given TB Preventive Therapy (TPT).^26^ On completion of TPT, BCG vaccination is advised, if no *M. tuberculosis* infection is demonstrated.

All infants in our cohort were clinically assessed at six weeks, as well as at six and 12 months of age. These clinical assessments were scheduled for the same day that the mother had to return for her monthly medical assessment and collection of pills. At the six-week visit, details including birth weight and whether the infant had been screened for TB at birth were collected from the *Road to Health* card (a booklet issued to mothers at the birth of their infant documenting weight, height and vaccination status of the infant). In addition, we also checked if HIV-exposed infants had been screened for HIV and given nevirapine prophylaxis, the routine standard of care in South Africa. At each of the three clinical visits all infants were monitored for growth and developmental milestones, as well as the development of any signs and symptoms of TB. These included ‘any one of the following: ‘cough for more than two weeks, fever for more than two weeks or poor weight gain in past three months, or chest radiograph or both’.^27^ It is recognised that symptom-based approaches have sub-optimal sensitivity for screening, especially with prespecified duration.^28^ At the six-week visit all infants had a chest radiograph taken and if there were changes suggestive of TB, a gastric aspirate was taken. At subsequent visits only infants who had developed signs and symptoms of TB were referred for a chest radiograph and gastric aspirate. All infants started on TB treatment during the follow up period were regarded as having TB disease.

### Data analysis

In the current analysis, we provide detailed mother-infant case-pair descriptions. We identified all infants started on treatment for TB following either a laboratory or clinical diagnosis. Based on their case histories, a further two infants (case histories 1 and 7) were included. We interrogated potential risk factors and health system failures by comparing the infants’ date of birth and development of TB with the treatment history of the mother to determine: 1) how long the mother had been on treatment before the infant was born; 2) how long she had been on treatment before the infant developed TB; 3) the mother’s sputum culture status at delivery and when her infant developed TB; and 4) the mother’s adherence to MDR/RR-TB treatment and ART. Baseline maternal and birth characteristics were compared, using the Fishers non-parametric test where indicated.

### Definitions

MDR/RR-TB was classified as TB with genotypic or phenotypic resistance to rifampicin,^29^ and the revised WHO definitions of pre-XDR-TB and XDR-TB were used to characterise the resistance patterns of all women, irrespective of when they were enrolled. MDR-TB was classified as resistance to at least isoniazid and rifampicin. Pre-extensively drug-resistant (pre-XDR) TB was classified as MDR-TB with additional resistance to a fluroquinolone, and extensively drug-resistant (XDR) TB classified as MDR-TB with additional resistance to a fluoroquinolone and an additional Group A drug (bedaquiline or linezolid).^29^ Resistance pattern definitions are detailed in Supplementary Table S2.

In infants born to mothers with TB, infection may be acquired *in utero*, during delivery or postpartum. Because it is often challenging to establish the precise route of transmission, the composite term perinatal TB is often used in infants diagnosed with TB in their first 3 months of life.^30^ The term congenital TB refers to *in utero* acquisition of *M. tuberculosis* by haematogenous spread through the placenta and umbilical vein, with a primary focus in the liver. It may also include the ingestion and or aspiration of *M. tuberculosis* via infected amniotic fluid at birth, with a primary focus in the gut or lungs.^30^ Postnatal transmission typically occurs through the inhalation of airborne *M. tuberculosis* bacilli.^1^ *M. tuberculosis* infection and TB disease refer to consensus definitions.^31^^,^^32^

When performing the critical case history assessment, we categorised each mother-infant pair according to the most likely route of transmission. Factors taken into consideration included the length of time the mother was on effective treatment before her baby was born, her sputum *M. tuberculosis* culture status at the time of delivery and when the infant developed TB, documented postpartum TB exposure and the age of the child when TB was diagnosed - together with the basis of their diagnosis.

### Ethics

This study was approved by the South African Medical Research Council (SAMRC) Ethics Review Committee (EC017/6/2016) and the KwaZulu-Natal Research Committee. The informed consent included maternal consent for the use of data for research and publication.

### Role of the funding source

No funder had any role in the study design, data collection, analysis, interpretation, writing up or decision to submit the manuscript for publication.

## Results

### Overview of mother-infant pairs

**Fig. 1:**
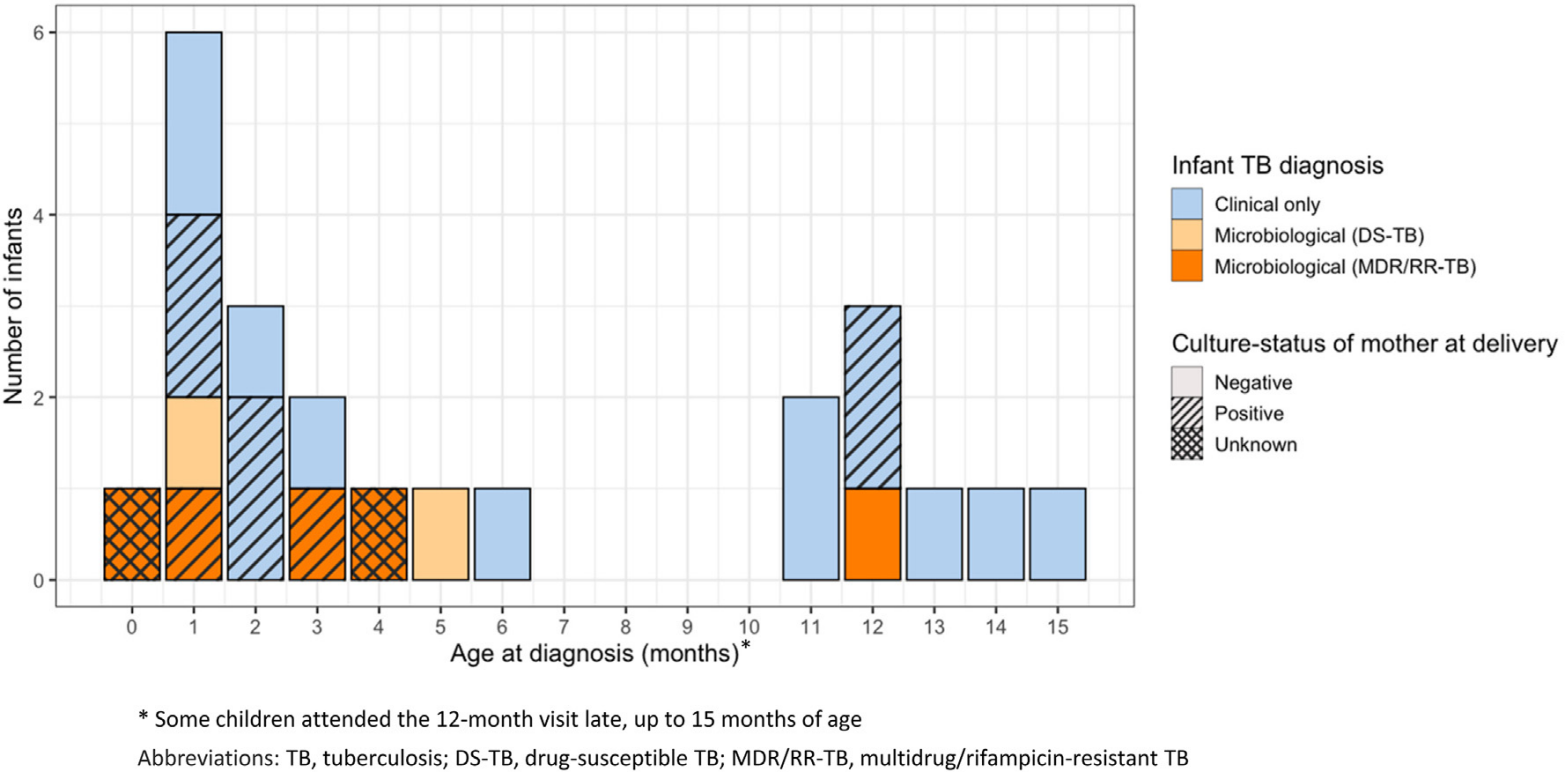
Timeline reflecting month, diagnostic method and maternal sputum culture status at the time of delivery in infants diagnosed with TB disease.

From 1 January 2013 to 31 December 2022, we enrolled 148 pregnant women with MDR/RR-TB. There were no multiple births (e.g., twins), 32 (22%) mother-infant pairs were lost to follow-up (LTFU), 13 (9%) had an unfavourable pregnancy outcome (stillbirth, miscarriage, early neonatal death or termination of pregnancy), and two (1%) infants died. Thus 101 women and their infants were available for longitudinal follow-up, whom we followed for at least 12 months. In total, 23/101 (23%) infants were diagnosed with TB; 12 within three months of life and three were diagnosed after the 12-month visit. The gap in diagnosis observed between months 6 and 11 reflects the timing of study visits. Fig. 1 illustrates maternal sputum culture status at birth and the age (in months) of infant TB diagnosis. Most were clinical MDR/RR TB diagnoses, but among those with microbiological confirmation two had fully drug-susceptible (DS-) TB and five had MDR/RR-TB.

Table 1 compares the clinical and birth characteristics of mothers with MDR/RR-TB whose infants were diagnosed with TB and those whose infants did not develop TB. Across the whole cohort, 84/101 (83%) mothers were living with HIV and 16/101 (16%) had pre-XDR- or XDR-TB. The baseline maternal and birth characteristics were similar, but significantly more mothers were sputum *M. tuberculosis* culture positive at birth (8/20; 40%) among the mother-infant pairs whose infants were diagnosed with TB, compared to pairs in whom the infants did not develop TB (10/70; 14%; p = 0.023). Of the 12 infants diagnosed with TB in the first three months of life, seven (66%) mothers were sputum culture positive at the time of delivery. In a sensitivity analysis comparing baseline clinical and birth characteristics of the 101 mother-infant pairs we followed-up for a year with the 32 mother-infant pairs LTFU, the differences did not appear to be significant (Supplementary Table S3). It is therefore unlikely that the exclusion of those LTFU would have changed our study findings. A LTFU rate of 20% in adult patients with MDR/RR-TB in our setting is normal.

**Table 1:** Clinical and birth characteristics in mothers with MDR/RR-TB whose infants were diagnosed with TB disease compared with those whose infants did not develop TB.

Baseline characteristics	Mother-infant pairs with infant TB (N = 23)	Mother-infant pairs without infant TB (N = 78)^a^
Age: years, median; [IQR]	28 [23–32.5]	28 [23–32.25]
HIV-positive: no (%)	18 (78%)	66 (85%)
Baseline CD4 count, median cells/mm3 [IQR]	410 [220–808]	410 [208–803]
**TB characteristics**		
Culture positive at TB treatment initiation	16 (70%)	(n = 76) 55 (72%)
Previous TB or MDR/RR-TB	(n = 19) 10 (53%)	(n = 66) 33 (50%)
Chest radiograph	(n = 22)	(n = 68)
Extensive disease pattern on chest radiograph^b^	9 (41%)	28 (41%)
Resistance pattern: no (%)		
RR-/Rif-mono/MDR-TB	19 (83%)	66 (85%)
Pre-XDR-/XDR-TB	4 (17%)	12 (15%)
Culture positive at delivery	(n = 20) 8 (40%)	(n = 70) 10 (14%)
Time from treatment initiation to delivery, median; [IQR]	116 [75–202]	114 [79–202]
**Birth characteristics**		
Gestational age at the time of maternal TB diagnosis: weeks, median [IQR]	24 [15.86–31.86]	24 [15.89–31.39]
Gestational age at delivery: weeks, mean; SD	36.78; 5.01	39.96; 4.44
Birth weight, grams, median [IQR]	2900 [2046–3400]	2900 [2455–3390]

no, number; IQR, interquartile range; HIV, human immunodeficiency virus; TB, tuberculosis; RR-TB, rifampicin-resistant TB; Rif-mono, rifampicin mono-resistant TB; MDR/RR-TB, multidrug/rifampicin-resistant TB; Pre-XDR-TB, pre-extensively drug-resistant TB; XDR-TB, extensively drug-resistant TB; SD, standard deviation.

a Variable N, as specified, excluding missing data.

b Bilateral lung disease and/or cavities.

Table 2 provides detailed clinical information of all infants diagnosed with TB. The median time from maternal TB treatment initiation to delivery was 116 days (IQR 75–202), but five mothers were on treatment for less than 2 months at the time of delivery. One mother (case history 8) was on treatment for only three weeks before she delivered but was culture negative at the time of delivery and fully adherent to treatment. However, her treatment adherence faltered postpartum, and her infant developed TB at 14 months of age. During the 12-month mother-infant follow-up period, we lost contact with two mothers of infants diagnosed with TB. One infant was left with his father and the second with her grandmother; according to the relatives, both mothers were still alive. Of the 21 mothers with whom we were able to maintain contact for a year, successful treatment outcomes were reported in eight; two failed to respond to treatment, four restarted treatment in their infant’s first year of life after initial poor adherence, three of whom were subsequently cured, while seven were lost to follow up after documented poor adherence.

**Table 2:** Demographic and clinical details of mother-infant pairs, where the mother had MDR/RR-TB and the infant was diagnosed with TB disease.

ID	Mother							Infant			
	Age	HIV status	CD4 count at TB treatment initiation	Drug resistance pattern	Initial TB treatment regimen^c^	Days from TB treatment initiation to delivery	TB culture status at birth	Gestational age (weeks)	Weight (grams)	Basis of TB diagnosis	Age at TB diagnosis (months)
CH 1	37	Negative	NA	MDR/RR-TB	Long	161	Positive	28	1800	Clinical	1
CH 2	22	Positive	9	MDR/RR-TB	Long	34	Positive	39	3120	Clinical	1
CH 3	26	Negative	NA	Pre-XDR-TB	Long	91	Positive	40	3600	Clinical	2
CH 4	38	Positive	51	XDR-TB	Long	38	Positive	38	2660	Clinical	2
CH 5	28	Positive	421	RR-TB	6-month	0^a^	Unknown	37	2840	Lab: MDR-TB	0
CH 6	29	Positive	384	XDR-TB	Long	383	Positive	31	1300	Lab: MDR-TB	1
CH 7	23	Positive	373	MDR/RR-TB	Short	153	Positive	40	3080	Clinical	6
CH 8	23	Negative	NA	MDR/RR-TB	Long + Inj	21	Negative	41	3600	Clinical	14
CH 9	35	Positive	15	Rif mono TB	Long + Inj	93	Positive	35	2820	Lab: MDR-TB	3
CH 10	19	Negative	NA	MDR/RR-TB	6-month	0^a^	Unknown	39	2920	Lab: MDR-TB	4
CH 11	18	Negative	NA	RR-TB	Long + Inj	53	Negative	39	2700	Lab: DS-TB	1
CH 12	38	Positive	340	Rif mono TB	Long	106	Negative	37	2250	Lab: DS-TB	5
ID 1	34	Positive	91	MDR/RR-TB	Short	240	Negative	39	2170	Clinical	1
ID 2	21	Positive	850	MDR/RR-TB	Long	585	Negative	36	2600	Clinical	1.5
ID 3	26	Positive	374	RR-TB	Long	152	Negative	38	2240	Clinical	2
ID 4	23	Negative	NA	XDR-TB	Long	144	Positive	34	1800	Clinical	3
ID 5	29	Positive	265	MDR/RR-TB	Long + Inj	593	Negative	39	3000	Clinical	11
ID 6	29	Positive	355	MDR/RR-TB	Long + Inj	190	Negative	40	3700	Clinical	11
ID 7	30	Positive	351	MDR/RR-TB	Long + Inj	22	Negative	40	2800	Lab: MDR-TB^b^	12
ID 8	29	Positive	424	MDR/RR-TB	Long	282	Negative	40	3900	Clinical	12
ID 9	24	Positive	429	MDR/RR-TB	Long	110	Unknown	39	2950	Clinical	12
ID 10	26	Positive	44	Rif mono TB	Short	113	Negative	42	2800	Clinical	13
ID 11	30	Positive	682	Rif mono TB	Long	53	Negative	39	3000	Clinical	15

CH, case history; ID, identification number; HIV, human immunodeficiency virus; TB, tuberculosis; MDR/RR-TB, multidrug/rifampicin-resistant TB; Pre-XDR-TB, pre-extensively drug-resistant TB; XDR-TB, extensively drug-resistant TB; RR-TB, rifampicin resistant TB; Rif mono TB, rifampicin mono-resistant TB; DS-TB, drug-susceptible TB; Lab, laboratory; Inj, injectable.

a Maternal MDR/RR-TB detected antenally, but only appreciated post-partum. When the infants were diagnosed with MDR/RR-TB, clinicians at King Dinuzulu Hospital searched the laboratory data base for maternal antenatal results and identified bacteriologically confirmed maternal MDR/RR-TB only after the infant was diagnosed and initiated on treatment.

b The infant was diagnosed with MDR/RR-TB based on a GeneXpert, and treated with the same regimen as the mother, on the assumption that she is the likely source.

c Details of the different MDR/RR-TB regimens used are reflected in Supplementary Table S1.

### Categorisation by likely route of *M. tuberculosis* transmission

We categorised each mother-infant pair according to the most likely route of transmission, considering all available information, with sub-categories reflecting the likely infectious source and key risk factors for infant disease development (Table 3).

**Table 3:** Individual description of mother-infant pairs in infants born to mothers with MDR/RR-TB, according to the infant’s likely *M. tuberculosis* transmission category.

Transmission category	Case histories
A. Likely *in utero* transplacental infection	*Case history 1* This HIV negative woman had a much awaited and longed for infant following in vitro fertilisation (IVF). The mother was diagnosed with miliary MDR/RR-TB^a^ at 5 weeks of gestation and started an all-oral 20-month regimen^b^ 5 days later. The baby was born 161 days after maternal treatment initiation at 28 weeks gestational age (GA), weighing 1.8 kg. At birth there were placental granulomas suggestive of congenital TB (the results could not be located) and the mother was sputum culture positive. The baby was hospitalised for a month with signs and symptoms of TB, as well as chest x-ray changes suggestive of TB disease. This infant was likely infected *in utero* and born with congenital MDR/RR-TB. Despite strong advice, the mother declined treatment of her infant, who continues to be monitored. The mother successfully completed treatment.
B. Possible intra-partum aspiration (difficult to exclude postpartum inhalation)	*Case history 2* At the time of her pregnancy, this mother lived in the same house as her mother (the infant’s grandmother) who had MDR/RR-TB. Living with HIV, the pregnant woman disengaged from care and did not take ART during the third trimester of her pregnancy. She developed MDR/RR-TB at 35 weeks gestation when her CD4 count was 9 cells/mm3. She was initiated on the all-oral 20-month regimen 34 days before her infant was born at 39 weeks GA, weighing 3,1 kg. At the time of delivery, the mother was not virally suppressed, (viral load 71577 copies/ml), and was sputum smear and culture positive. A week later the infant was admitted to the local hospital where, despite the gastric aspirate being negative for *M. tuberculosis*, there were changes on chest x-ray. As the infant failed to respond to antibiotics, MDR/RR-TB was considered the most likely diagnosis, possibly due to intra-partum aspiration. The infant was started on the 9-month MDR/RR-TB regimen 12 days after birth. Both the mother and infant responded to treatment which they successfully completed. *Case history 3:* This HIV-negative mother was diagnosed with pre-XDR-TB^c^ at 27 weeks of gestation and started on an all-oral 20-month regimen. Her infant was born 91 days later at 40 weeks GA weighing 3.6 kg, when the mother was still sputum smear and culture positive. The infant developed signs and symptoms of TB (fever, cough, and chest x-ray abnormalities) at 6 weeks of age and was referred to the local clinic for a gastric aspirate collection. Although the gastric aspirate was negative, it was assumed the infant had MDR/RR-TB and he was started on a 9-month regimen based on his mother’s resistance pattern. *M. tuberculosis* transmission was possibly due to intra-partum aspiration, although the possibility of postpartum inhalation cannot be excluded. By the 6-month visit the infant’s TB signs and symptoms had fully resolved and by the 12-month visit was picking up weight. The mother subsequently successfully completed treatment. *Case history 4:* This mother living with HIV was diagnosed with XDR-TB^d^at 32 weeks gestation when her CD4 count was 51 cells/mm3. She started on an all-oral 20-month regimen the following day but stopped taking treatment at 36 weeks gestation. Her infant was born at 38 weeks GA weighing 2.7 kgs. At the time of delivery, the mother was sputum culture positive. She reinitiated TB treatment when her infant was 4 weeks old, but at 8 weeks old the baby started losing weight and coughing. Her chest x-ray showed right upper lobe infiltrates with left hilar prominence. Although the gastric aspirate was negative, MDR/RR-TB treatment based on the mother’s resistance pattern, was initiated. It is possible that intra-partum aspiration was responsible for this infants perinatal TB, although postpartum inhalation cannot be excluded. The infant responded well to treatment, completed the full 9-month course and was discharged. The mother failed treatment and was restarted on a ‘rescue regimen’ which she did not complete.
C. Likely post-partum inhalation	
C.1. Likely maternal source	
C.1.1. Infectious mother not provided with appropriate treatment	*Case history 5* This mother living with HIV first attended antenatal care at 29 weeks gestation. In line with country guidelines, she was tested for TB and started on DS-TB treatment. However, the health facility failed to pick up that she had MDR/RR-TB. Her baby was born two months later at 37 weeks gestational age, weighing 2.8 kg. The baby was screened for TB at birth. Given that the TB diagnosis was made soon after birth, with perihilar changes on chest x-ray, no sign of a Ghon focus in the liver and a gastric aspirate positive for MDR/RR-TB on Xpert MTB/RIF, this was considered likely intra-partum aspiration. Both the mother and infant were started on a 9-month treatment regimen based on the mother’s resistance pattern within the first few days of the infant’s life, responding well to treatment and were cured and completed treatment respectively. *Case history 6* This mother living with HIV fell pregnant whilst on MDR/RR-TB treatment. She was initially tested with Xpert MTB/RIF and although a subsequent culture and drug susceptibility testing (DST) showed pre-XDR-TB with fluoroquinolone resistance, she was treated with a fluoroquinolone containing regimen. She fell pregnant on treatment, but at 23 weeks gestation after 318 days of treatment, was still culture positive. Acid-fast bacilli (AFB) were detected on sputum smear microscopy and her DST showed additional resistance to bedaquiline and clofazimine. She was started on an all-oral 20 month ‘rescue regimen’ at 29 weeks gestation and her infant was born two weeks later at 31 weeks GA age weighing 1.3 kg. At the time of delivery the mother’s viral load was 23 copies/ml and her CD4 count 384 cells/mm3, she was still sputum culture positive, but as the baby was not considered to have TB disease was discharged. It is not clear whether this infant was given TB Preventive Therapy (TPT) as it was not recorded in the Road to Health card. At two weeks of age the infant was admitted to hospital with respiratory distress. As her chest radiograph showed extensive reticulo-nodular infiltrates and MDR/RR-TB was detected on her gastric aspirate, it is likely this infant was infected by intrapartum aspiration. The infant responded well to a 9-month regimen based on the mother’s resistance pattern and having completed the course was discharged. The mother subsequently completed treatment.
C.1.2. Infectious mother with poor treatment adherence	*Case history 7:* This mother was diagnosed with MDR/RR-TB and started an all-oral 9–11 months regimen at 18 weeks gestation. At the same time, she was diagnosed as HIV-positive and started ART two weeks later. She took treatment for 66 days and then stopped both her TB treatment and ART. Three months later, when she was still sputum culture positive, her baby was born at 40 weeks gestation weighing 3.08 kg. The mother started taking treatment (TB treatment and ART) again when her infant was 2 months old, but only for a short time. She disengaged from care and at 5 months left her infant in the care of his father. The infant was not brought for his scheduled 6-month visit, but after repeated calls was brought in at 9 months. He was very unwell, with a fever, cough, losing weight, and a chest x-ray showing extensive changes suggestive of TB. He was referred for a gastric aspirate, but this was never taken. We were informed he died 2 months later. It is likely this infant was infected postnatally due to sub-optimal maternal adherence to both MDR/RR-TB treatment and ART. We lost all contact with the mother and cannot report her outcome. *Case history 8:* This mother, who was HIV-negative was diagnosed with MDR/RR-TB and started on the 18-24-month regimen with an injectable agent at 37 weeks gestation. Her infant was born 3 weeks later at 41 weeks gestation weighing 3.6 kg. At the time of delivery, the mother was sputum smear and culture negative. The mother stopped taking TB treatment when her infant was three months old. Her infant developed signs and symptoms of TB (cough, lethargy, losing weight with chest radiography changes suggestive of TB) at 14 months of age, but a gastric aspirate done at the time was negative. It is likely the infant was infected postnatally due to sub-optimal maternal adherence to both MDR/RR-TB treatment and ART. The child was started on the same regimen as the mother, but unfortunately the mother and infant were lost to follow up.
C.1.3. Infants not screened for TB or provided with appropriate TPT at birth	*Case history 9:* This mother living with HIV, was poorly adherent to her ART and diagnosed with MDR/RR-TB at 22 weeks gestation and started on a 20-month regimen with an injectable 2 months later, with a CD4 count of 15. Three months after starting TB treatment, at the time of delivery, her sputum smear and culture were still positive. Her infant was born premature at 35 weeks gestation weighing 2.82 kg. TB screening at birth was negative, but the infant was not given TPT or a BCG vaccination. At 3 months of age the infant developed signs and symptoms of TB (cough and losing weight) and a gastric aspirate detected MDR/RR TB on Xpert. A long treatment regimen with an injectable was initiated. Given poor maternal treatment adherence, sputum culture positivity at delivery and the absence of any other household members with infectious TB, it is likely that the mother infected her infant post-partum with a very high risk of disease progression in the absence of BCG or TPT. The mother and infant were cured and completed treatment respectively.
C.2. Likely non-maternal source	
C.2.1. Non-maternal source inside the household	*Case history 10:* The father of this infant was diagnosed with MDR/RR-TB early in the mother’s pregnancy (12 weeks gestation). He was initially started on an all-oral 9-month regimen, but as he failed to respond to treatment, was changed to a long regimen all-oral regimen a month before the infant was born. The mother, who was HIV negative, was not screened for TB during antenatal care and neither she nor the infant were screened for TB when the infant was born at 39 weeks gestation weighing 2.92 kgs. At 4 months old the infant developed signs and symptoms suggestive of TB (cough, fever and loss of weight). His gastric aspirate was cultured with a similar phenotypic resistance pattern to that of his father and he was started on a 9-month treatment regimen. When he was 6 months old his mother also developed MDR/RR-TB with the same resistance pattern as the father and started on an appropriate 9-month regimen. It is likely this infant and his mother were infected by his father. Both the mother and infant are responded well to treatment which is now complete. *Case history 11:* This mother was HIV-negative. She was diagnosed with MDR/RR-TB in March 2016 at 31 weeks gestation and started on a 20-month regimen with an injectable. Her baby was born 8 weeks later at 39 weeks gestation weighing 2.7 kg. The mother was sputum smear and culture negative at the time of delivery. The infant was diagnosed with laboratory confirmed drug susceptible (DS) TB in the first month of life. Five other people were living in the same house as the mother-infant pair, one of whom the father was on DS-TB treatment. Given that the infant was only 1-month old when she developed TB symptoms (fever, cough, and lethargy), she was unlikely to have been infected outside the household. It may have been that her father had sub-optimal adherence, or another household member may have had TB that was undisclosed or not yet diagnosed. There is a possibility that the mother might have had a mixed (multi-strain) infection but was never microbiologically confirmed. The infant responded well to treatment and completed the full 9-month course. The mother stopped treatment after 10 months and although we saw her at her infants 12-month follow-up visit, we are unsure of her health status, as we lost contact hereafter.
C.2.2. Non-maternal source outside the household	*Case history 12:* This mother living with HIV was diagnosed with MDR/RR-TB at 20 weeks gestation and started a 9-month all-oral regimen treatment 3 days later. Her infant was born at 37 weeks gestation 3.5 months later, weighing 3.5 kg. At the time of delivery, the mother was sputum smear and culture negative, but had stopped taking both MDR/RR-TB treatment and ART, which she only restarted her infant was diagnosed with culture-confirmed DS-TB at 5 months old. Given that this infant was 5 months old it is possible that transmission may have occurred outside the household. However, all possibilities need to be considered and given that the mother’s adherence was sub-optimal she could have been infectious and infected her baby with a DS-TB strain if she had a mixed infection. Additional TB exposure within or outside the household was undocumented and is difficult to verify. Routine whole genome sequencing of all patients with infectious TB, as practiced in some low-incidence settings may have helped to clarify the likely transmission chain, but this is not done in South Africa.^30^

IVF, in vitro fertilisation; TB, tuberculosis; MDR/RR-TB, multidrug/rifampicin-resistant TB; GA, gestational age; ART, antiretroviral therapy; chest x-ray, chest radiograph; pre-XDR-TB, pre-extensively resistant TB; XDR-TB, extensively drug-resistant TB; DS-TB, drug-susceptible TB; DST, drug susceptibility testing; AFB, acid-fast bacilli; TPT, TB preventive therapy; BCG, Bacillus Calmette-Guérin.

a MDR/RR-TB was classified as TB with resistance to rifampicin mono-resistant tuberculosis (susceptibility to isoniazid), and forms of disease where rifampicin resistance has been identified, but no result for isoniazid is available.^1^ It includes MDR-TB which is classified as resistance to isoniazid and rifampicin.

b Details of the different MDR/RR-TB regimens are in Supplementary Table S2.

c Pre-XDR-TB was classified as TB that met the definition of MDR/RR-TB with additional resistance to any fluroquinolone. The definitions of pre-XDR-TB and XDR-TB that were used are detailed in Supplementary Table S1.

d XDR-TB was classified as TB that met the definition of MDR/RR-TB with additional resistance to at least one fluoroquinolone (levofloxacin or moxifloxacin) and at least one additional Group A drug (bedaquiline or linezolid).

The following possible routes of transmission were considered: 1) *in utero* transplacental infection (case history 1); 2) intra-partum aspiration (case histories 2, 3, and 4); and 3) postpartum inhalation. Postpartum inhalation was divided into likely maternal vs. non-maternal sources of infection. In infants with a likely maternal source of infection we considered: 1) mothers with sub-optimal treatment (case histories 5 and 6); 2) mothers with sub-optimal treatment adherence (case histories 7 and 8); and 3) infants not screened for TB or provided with appropriate TPT (case history 9). A likely non-maternal source of infection was identified within (case histories 10 and 11) and outside the household (case history 12). Of the 12 infants who developed TB in the first three months of life, seven mothers were sputum culture positive at delivery, of whom four reported poor TB treatment adherence. Of the 13 infants who developed TB after three months of age, a further six mothers reported sub-optimal adherence at some point during their treatment journey.

### Health system failures

Failures in the health system likely contributed to the development of TB in three infants. Two mothers were treated with an ineffective MDR/RR-TB regimen. The first mother (case history 6) was treated with a fluoroquinolone containing regimen, despite documented resistance to fluoroquinolones. The second mother (case history 5) was tested for TB during antenatal care, but her positive MDR/RR-TB laboratory result was not acted upon, and she was started on inappropriate DS-TB treatment. In the third instance, the infant’s father was diagnosed with MDR/RR-TB during the mother’s pregnancy, but she was not started on TPT and after delivery no consideration was given to the household transmission risk posed to the infant and appropriate TPT was not provided prior to discharge home. After four and six months respectively, both the infant and then the mother were diagnosed with MDR/RR-TB that displayed the same resistance pattern as the father. Table 4 tabulates the number and proportion of infants screened for TB and managed according to the existing South African guidelines.^26^ In general, national guidelines were poorly implemented with limited recording or reporting of appropriate management. Although 58/101 (58%) infants were documented as having been screened for TB, 43 (42%) with a chest radiograph and 45 (44%) with a gastric aspirate, only 47 (46%) received TPT. In South Africa, isoniazid preventive therapy (IPT), dosed according to weight, is given to all infant contacts of MDR/RR- and DS-TB.

**Table 4:** At-birth management of infants born to mothers with MDR/RR-TB (N = 101) (based on 2019 South African guidelines used during study period).

Management component	Yes	Not done	Not recorded
Screened for TB at birth	58 (58%)	12 (12%)	31 (30%)
Chest radiograph done	43 (42%)	25 (25%)	33 (33%)
Gastric aspirate done	45 (44%)	20 (20%)	36 (36%)
TPT given at birth^a^	47 (46%)	22 (22%)	32 (32%)
BCG given at birth	36 (36%)	43 (42%)	22 (22%)
BCG given after TPT (N = 47)	22 (47%)	20 (43%)	5 (10%)

TB, tuberculosis; TPT, TB preventive therapy; BCG, Bacillus Calmette-Guérin.

a Isoniazid Preventive Therapy was the only TPT regimen available in South Africa during the study period.

## Discussion

In an operational setting with high burdens of TB, HIV and MDR/RR-TB, we describe 23 mother-infant pairs where the mother had MDR/RR-TB and her infant developed TB in the first year of life. The proportion of infants who developed TB was high with nearly a quarter of infants born to mothers treated for MDR/RR TB in pregnancy diagnosed with TB in their first year of life. The mothers’ sputum *M. tuberculosis* culture status at delivery was strongly associated with infant disease development. Sub-optimal maternal treatment adherence was identified in the majority of mothers who were culture positive at the time of delivery, while several health system failures, including failure of MDR/RR recognition and treatment, also contributed to the infant’s TB disease risk.

The high TB disease rate among infants born to mothers with MDR/RR-TB, with most developing TB disease within the first three months of life, is consistent with previous findings from the same area where an infant disease rate of 16% was reported in 107 pregnant women with TB.^33^ Although this 2004 study was conducted when ART eligibility was limited to those with a CD4 < 200, the risk of *M. tuberculosis* transmission from mother to infant was independent of maternal HIV infection or CD4 count. Infants are extremely vulnerable to TB and can be equally affected by drug resistant and drug susceptible strains of *M. tuberculosis.* Apart from infants’ high risk of progressing from *M. tuberculosis* infection to disease, they also experience the highest risk of any age group of developing severe disseminated forms of disease, such as miliary TB and tuberculous meningitis, with high TB-related mortality.^34^

Of the 12 infants who developed TB in the first three months of life, over half (7/12; 58%) of the mothers were sputum culture positive at delivery, of whom four reported poor adherence prior to, during and after delivery. Of the 13 infants who developed TB after three months, six mothers reported sub-optimal adherence at some point during their treatment journey, and all were sputum culture positive at the time of the infants’ TB diagnosis. This is highly relevant, given that sub-optimal MDR/RR-TB and ART adherence can result in reversion to sputum culture positivity and infectiousness with possible transmission to the infant.^35^^,^^36^ In the study setting, pregnant and postpartum women co-infected with TB and HIV contend with numerous challenges to remain adherent and engaged in health care, given many distracting demands and limited control of their lives.^37^^,^^38^ In addition to increased financial challenges during the peri-partum period, women with MDR/RR-TB are particularly vulnerable to social isolation due to community stigma and fear, as well as discriminatory practices and sub-optimal care during antenatal care and delivery.^39^^,^^40^ Although adherence to MDR/RR-TB treatment in pregnancy and the postpartum period is essential, many drugs used in MDR/RR-TB regimens cause nausea,^41^ which may be exacerbated in pregnancy. Pregnant women have also reported morning sickness and physical discomfort as barriers to ART adherence in the antenatal period,^42^ although a systematic review and meta-analysis found that ART adherence was higher during pregnancy than in the postnatal period (76% vs 53%; p = 0.005).^43^ Postpartum barriers to treatment adherence include a dramatic change in daily routine, the demands of caring for a new baby, fatigue, sleep deprivation and postpartum depression.^42^^,^^44^

**Fig. 2:**
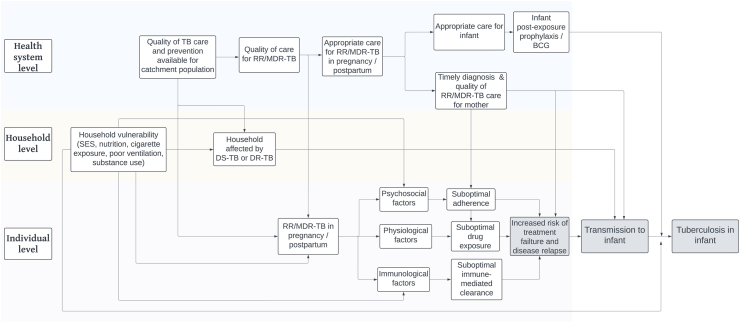
Conceptual framework illustrating the potential pathways leading to mother-to-child transmission of *M. tuberculosis* and tuberculosis in the infant.

It is generally accepted that a TB patient is no longer infectious after two weeks of effective treatment.^45^^,^^46^ However, there are limited data on the optimal use of second-line TB drugs in pregnant women,^47^ and the physiological changes in pregnancy may result in changes in TB drug absorption, distribution and clearance, impacting treatment efficacy.^48^, ^49^, ^50^, ^51^ In addition, in pregnant women with pregnancy related immunosuppression, increased social and emotional stress caused by pregnancy, as well as post-natal physical exhaustion and depression, *M. tuberculosis* excretion may be prolonged; even more so in mothers living with HIV.^52^ It is biologically plausible that infectiousness may be prolonged, due to the multiple immunological, physiological and social phenomena at play, potentially increasing the risk of *M. tuberculosis* transmission to the infant (Fig. 2).^53^^,^^54^ In addition there are multiple factors that make infants particularly vulnerable to develop TB disease.^55^

Health system failures also require critical evaluation. In South Africa, all infants born to mothers with TB are supposed to be screened for TB with initiation of appropriate TPT if active disease is excluded. In the study setting, less than half of the infants were screened for TB at birth and even less were provided with any form of TPT. More appropriate TPT should become available given the recent release of updated WHO TPT guidelines for MDR/RR-TB, recommending six months of levofloxacin for all MDR/RR-TB contacts, including infants, exposed to a patient with infectious MDR/RR-TB.^56^ Given the high risk of infants born to mothers with TB,^53^^,^^57^^,^^58^ there is a global push to increase TPT coverage, especially of the most vulnerable young children.^12^ However, the response has been modest in many high TB burden low and middle income countries and systematic and consistent implementation is deficient due to a lack of pragmatic decentralised strategies, resource constraints and limited monitoring and evaluation.^59^, ^60^, ^61^ In addition, healthcare workers are often hesitant to provide TPT in the absence of clear and consistent guidelines. They fear adverse drug reactions and the amplification of drug resistance, although this is irrelevant in vulnerable young children.^62^ Since only isoniazid preventive therapy (IPT) is currently available in most settings, many healthcare workers are reluctant to provide IPT, which they believe to be ineffective, to household contacts of a person with MDR/RR-TB. This should change with access to levofloxacin TPT, which has proven efficacy^56^ and is available as child-friendly formats. Studies to assess the safety and efficacy of pragmatic screening tools, optimal TPT regimens to use in infancy and specific strategies to limit postpartum transmission are needed.^63^^,^^64^ In addition, the development of diagnostic tools for use in newborns and infants, using specimens other than sputum, are urgently needed to ensure newborns with perinatal TB are correctly diagnosed and TB disease excluded before starting TPT.^65^^,^^66^

In many settings TB and HIV services are not aligned with maternal and child health services. In the study setting, mothers living with HIV and their infants were expected to attend the clinic up to six times in the infants first six months of life (TB and HIV clinic visits for picking up repeat medication and monitoring response to treatment were scheduled on a 28-day pill cycle, whereas postnatal and child growth monitoring and vaccination clinic visits were scheduled by week or month.)^67^ This resulted in missed clinic visits and poor postpartum ART and TB treatment adherence as well as repeated exposure of infants to the risk of *M. tuberculosis* transmission, both *en route* to the clinic in crowded taxis and in the clinic. Luckily this has now been addressed with better coordination of TB, HIV and routine postnatal care. Table 5 provides an overview of service delivery challenges identified and summarises recommendations for minimising *M. tuberculosis* transmission and disease risk in infants born to mothers with TB, drawing on key study findings.^40^^,^^56^^,^^64^^,^^68^^,^^69^

**Table 5:** Identified health service challenges and suggested recommendations to minimise *M. tuberculosis* transmission from mothers with TB to their infants.

Health service challenge	Recommendations
Absence of routine screening for TB in the ante- and post-partum periods	In high TB incidence settings pregnant women should be routinely screened for TB at every ante- and post-partum visit. If TB disease is ruled out, they should be considered for TPT. Although an increased risk of adverse pregnancy outcomes has been reported in a small study in women exposed to IPT in the first trimester,^68^ a study in over 43,000 pregnant women in our setting reported that women who received IPT were less likely to experience adverse pregnancy outcomes and IPT reduced the risk of TB by 30%.^69^ Women of reproductive age who develop TB should be advised to avoid falling pregnant and provided with contraceptives, while on TB treatment.
Poor integration of TB into primary maternal and child health services.	TB services must be fully integrated into maternal and child health services, to reduce the number of clinic visits during the stressful postpartum period. Pregnant/post-partum women and their vulnerable infants should not sit in overcrowded and poorly ventilated clinic waiting rooms, where there is a high risk of TB exposure. Healthcare workers in delivery wards must be trained to routinely screen infants for TB as is locally feasible if their mother has TB,^64^ and to provide TPT or BCG as appropriate with encouragement of treatment adherence.
Infant TPT	The updated World Health Organization TPT guidelines for MDR/RR-TB, recommending six months of levofloxacin for all contacts of infectious MDR/RR-TB patients, including infants, will soon be available.^56^ The availability of child-friendly dispersible levofloxacin should facilitate infant administration, reducing the risk of TB disease development in infants exposed to mothers with MDR/RR-TB.
Suboptimal maternal TB and anti-retroviral treatment adherence.	Additional counselling, information and adherence support for women on TB, or MDR/RR-TB treatment and ART should be provided during pregnancy and the postpartum period. In the third trimester, this should include the importance of adherence over the time of delivery and careful preparation for this, together with multi-month scripting, in case the delivery coincides with the next appointment. Health care workers providing antenatal and delivery services must be educated about the importance of uninterrupted MDR/RR-TB treatment during pregnancy and delivery. Appreciating that this is highly effective in reducing infectiousness and keeping health care workers safe, should also help to minimise discriminatory practices which contribute to poor adherence.^40^ Should a woman disengage from care, she should be traced and welcomed back into care on returning to a health facility. Future qualitative studies are needed to understand the challenges to adherence pregnant and postpartum women experience.

TB, tuberculosis; TPT, TB preventive therapy; IPT, isoniazid preventive therapy; MDR/RR-TB, multidrug/rifampicin-resistant TB; ART, antiretroviral therapy.

Several study limitations must be acknowledged. This was a pragmatic observational study conducted in the public sector, using routinely collected data that were often missing or incomplete. Initially data on parity was not collected. Furthermore, data on time to positivity for maternal cultures was not collected, which may have provided an additional measure of ‘bacterial load’ and degree of infectiousness. Data on adherence was collected in discussion with the mothers, and although we got to know the mothers well and could have frank discussions, we had no objective measure of treatment adherence. The study was limited in size and performed at a single centre that may not be representative of challenges experienced elsewhere. It should be acknowledged that the lack of tests done to confirm *in utero* transplacental infection and or intra-partum aspiration complicated the categorisation of the likely route of transmission. This was compounded by our limited resources, as there was no capacity to do genotyping to compare the *M. tuberculosis* strains isolated in mothers, infants, and household contacts. We therefore categorised mother-infant pairs according to the most likely route of infection, from the information available. However, despite these limitations it is the largest study of maternal/infant pairs in mothers treated for MDR/DR TB during pregnancy and the experience documented should be highly representative of challenges in other high TB incidence settings with high rates of HIV infection.

Given the increased risk of TB in pregnant and postpartum women and the severe consequences of *M. tuberculosis* transmission to their infants, it is critical to consider how maternal TB care can be optimised and infants, together with the rest of the family protected against TB development, especially MDR/RR TB. Key challenges that need to be addressed include: 1) the absence of routine screening for TB in the ante- and post-partum periods in high TB incidence settings; 2) infant TPT; 3) poor integration of TB into primary maternal and child health services; and 4) suboptimal maternal TB and ART adherence during the disruptive peri-partum period.

## Contributors

ML, NG, PK, KH, GT and BM conceptualised the study and with KH, SC and NS designed the study and participated in consensus deliberations. ML, SH, KH and SC collected and curated the data. ML, KH and BM accessed and verified the data. ML, SH, KH, SC, NG, PK, GT and BM conducted the data analysis. ML and BM wrote the original draft. All authors contributed expert knowledge, critically reviewed and edited the manuscript and approved the final version.

## Data sharing statement

The study materials and de-identified datasets can be requested by qualified researchers for use in scientific research. Sharing of data is subject to protection of patient privacy and respect for the patient’s informed consent. The data will be provided following review and approval of a research proposal and execution of a Data Sharing Agreement.

## Declaration of interests

We declare no competing interests.
