# Supplementary material for: Critical assessment of infants born to mothers with drug resistant tuberculosis
Source: eClinicalMedicine. 2024 Sep 5;76:102821. doi: 10.1016/j.eclinm.2024.102821 (PMC11405821; doi:10.1016/j.eclinm.2024.102821)
Supplement: Supplementary Tables S1–S3 [file mmc1.docx]

**Supplementary Table 1: The history of MDR/RR-TB treatment for pregnant women and children in South Africa***

| **Year** | **Regimen** |
| --- | --- |
| Prior to 2015 | All people with MDR/RR-TB treated with 18 – 24-month regimen which included an injectable in accordance with the WHO 2011 treatment guidelines:   - 6 - 8 months injectable phase: Kanamycin (KM), moxifloxacin (MFX), ethionamide (ETO), terizidone (TRD) and pyrazinamide (PZA) - 12 – 18 months: MFX, ETO, TZD, PZA^1^   In some patients ethambutol (EMB) and higher doses of isoniazid (hINH) were added.  **In pregnant women** the injectable and ETO were omitted, stopped or substituted with alternate agents.  **Children** received the same regimen as detailed above. |
| 2015 | All people with MDR/RR-TB in South Africa (including pregnant women) had access to bedaquiline (BDQ). |
| 2017 | WHO shortened regimen (9 – 12 months) introduced in South Africa, with either an injectable or BDQ:^2^  KM or BDQ + clofazimine (CFZ) + a fluoroquinolone (FQ) + ETO + hINH + EMB + PZA  **In pregnant women** the injectable and ethionamide were omitted, stopped or substituted with alternate agents.  **Children** continued to be treated with the WHO 2011 regimen as BDQ was only recommended for the treatment of children > 6 years. |
| 2019 | **All oral regimens:^3^**  ***9 – 11-month short regimen:*** *(for minimal disease)*   - 4 – 6 months intensive phase: Linezolid (LZD) for 2 months only + BDQ +hINH + levofloxacin (LFX) + CFZ + PZA + EMB - 5 months continuation phase: LFX + CFZ + PZA + EMB   ***Basic 18 - 20-month long regimen***:   - 6 months intensive phase: BDQ+ LZD + LFX + CFZ + TRD (If FQ resistant DLM) - 12 months continuation phase: LFX + CFZ + TRD (and/or Bdq and/or DLM if one of the other drugs is not tolerated.   ***Longer regimen for fluoroquinolone-resistant MDR/RR-TB***  ***18 – 20-month basic long regimen:*** *(for patients with extrapulmonary disease, both Inh mutations (inhA and katG), previous MDR-TB or extensive disease)*   - 6 – 8 months intensive phase: BDQ + LZD + DLM + CFZ + TRD - 12 months continuation phase: CFZ + TRD + BDQ (and/or DLM depending on tolerance to other drugs)   Pregnant women together with others with MDR/RR-TB were eligible to receive the shorter 9–11-month regimen if they met the inclusion criteria: no prior history of treatment with second-line TB drugs (>1 month); no evidence of resistance to fluoroquinolones, bedaquiline, clofazimine or linezolid; only one INH mutation (*inhA* or *katG*) or no mutation causing isoniazid resistance; no close contact with individuals with the above-mentioned characteristics; no evidence of complicated extra-pulmonary MDR/RR-TB (i.e. meningitis, pericarditis, osteoarticular, abdominal disease) or extensive, bilateral, cavitary pulmonary disease.  ***In children ≤ 3 years of age:***  Children with non-severe disease can be treated for 9 to 15 months, while children with severe disease will require 12 to 18 months treatment depending on their clinical progress.   - Regimen for FLQ sensitive disease: LFX + LZD + CFZ + TRD (and or para-aminosalicylic acid (PAS) or ETO/hINH depending on drug tolerability) - Regimen for FLQ resistant disease: LZD + CFZ + TRD +DLM (and PAS and/or ETO/hINH depending on drug tolerability) |
| 2023 | On 1 September 2023 the South African equivalent of the 6-month BDQ + Pretomanid (Pa) + LZD + Moxifloxacin regimens were rolled out (BPaLL or BPaL).^4^ In South Africa levofloxacin is being used instead of moxifloxacin. Patients susceptible to fluoroquinolones will receive: BDQ + Pa + LZD + LFX (BPaLL). Patients resistant to fluroquinolones will receive a 3-drug regimen: BDQ + Pa + LZD (BPaL)  ***Pregnant women:*** Due to uncertainty regarding the reproductive toxicity of pretomanid, pregnant women will receive delamanid instead of pretomanid:   - Pregnant women susceptible to fluoroquinolones will receive:   Bedaquiline + delaminid + linezolid + levofloxacin (BDLL)   - Patients resistant to fluroquinolones will receive a 3-drug regimen: Bedaquiline + delamanid + linezolid (BDL)   Pregnant women together with others with MDR/RR-TB who have extensive pulmonary disease (i.e., bilateral, cavitary with significant fibrosis, scarring or cavities in 3 or more lung zones) have their treatment extended to 9 months.  Pregnant women together with others with MDR/RR-TB who fail treatment with the 6-month regimen, have severe extra-pulmonary disease (meningitis, pericarditis, osteoarticular, abdominal or disseminated/miliary disease) are treated with a long-individualised regimen.  ***Children < 15 years:***  Children who are susceptible to fluoroquinolones:  Non-severe disease: BDQ + LFX + CFZ + (TRD or DLM) for 6 months; and +/- LZD for first 2 months  Severe disease: BDQ + LFX + LZD + CFZ + (TRD or DLM) for 9 months  Children who are resistant to fluoroquinolones:  Non-severe disease: BDQ + LZD + CFZ + TRD + DLM for 6 months  Severe disease: BDQ + LZD + CFZ + TRD + DLM for 9 months |

Abbreviations: MDR/RR-TB, multidrug/rifampicin-resistant TB; KM, kanamycin; MFX, moxifloxacin; ETO, ethionamide; TRD, terizidone; PZA, pyrazinamide; BDQ, bedaquiline; CFZ, clofazimine; FQ, fluoroquinolones; hInh, high dose isoniazid; Pa, pretomanid; EMB, ethambutol; LZD, linezolid; LFX, levofloxacin; DLM, delamanid.

* The regimens for children with central nervous system or miliary disease are not described as no infants in our cohort had these diseases.

**Supplementary Table 2: Definitions of MDR/RR-TB resistance patterns^4,5^**

| **Drug resistance definitions** | |
| --- | --- |
| Multidrug/rifampicin resistant TB (MDR/RR-TB) | MDR/RR-TB was classified as TB caused by *Mycobacterium tuberculosis (M.tuberculosis)* with genotypic or phenotypic resistance to rifampicin. It included MDR-TB (resistance to both isoniazid and rifampicin), rifampicin mono-resistant tuberculosis (susceptibility to isoniazid), and forms of disease where rifampicin resistance has been identified, but no result for isoniazid is available. |
| Pre-extensively drug-resistant TB (Pre-XDR-TB) | Pre-XDR-TB was classified as TB disease caused by strain of *M.tuberculosis* that meet the definition of MDR/RR-TB with additional resistance to any fluroquinolone. |
| Extensively drug-resistant TB (XDR-TB) | XDR-TB was classified as TB disease caused by strain of *M.tuberculosis* that meet the definition of MDR/RR-TB with additional resistance to at least one fluoroquinolone (levofloxacin or moxifloxacin) and to at least one additional Group A drug either BDQ or LZD. |

Abbreviations: MDR/RR-TB, multidrug/rifampicin-resistant TB; BDQ, bedaquiline; LZD/, linezolid

**Supplementary Table 3:** **Clinical and birth characteristics in mothers with MDR/RR-TB whose infants were followed up for 12 months compared to those who were lost to follow up and not included in our study.**

| **Baseline characteristics** | **Mother-infant pairs included in study**  **(N=101)** | **Mother-infant pairs lost to follow-up**  **(N=32)** |
| --- | --- | --- |
|  |  |  |
| Age: years, median; [IQR] | 28 [23-32·5] | 28 [23-32] |
| HIV-positive: no (%) | 84 (83%) | 26 (81%) |
| Baseline CD4 count, median cells/mm3 [IQR] | 410 [219-807·5] | 416 [219-809] |
| **TB characteristics** |  |  |
| Culture positive at TB treatment initiation | (N=99)  71 (72%) | (N=25)  17 (68%) |
| Previous TB or MDR/RR-TB | (N=85)  43 (51%) | (N=26)  12 (46%) |
| Chest radiograph: Extensive disease pattern on chest radiograph^†^ | (N=90)  37 (41%) | (N=31)  14 (45%) |
| Resistance pattern: no (%) |  |  |
| RR-/Rif-mono/MDR-TB | 85 (84%) | 26 (81%) |
| Pre-XDR-/XDR-TB | 16 (16%) | 16 (19%) |
| Culture positive at delivery | (n=90)  18 (20%) | N=16  4 (25%) |
| Time from treatment initiation to delivery, median; [IQR] | 114 [76·4-202] | 122 [77-219] |
| **Birth characteristics** |  |  |
| Gestational age at the time of maternal TB diagnosis: weeks, median [IQR] | 24 [15·88-31·45] | 24 [15·71-31·42] |
| Gestational age at delivery: weeks, mean; SD | 38·87; 4·94 | 38·.00; 4·24 |
| Birth weight, grams, median [IQR] | 2900 [2452-3400] | 2900 [2468-3400] |

**REFERENCES**

1. South African Department of Health. Management of drug-resistant tuberculosis. Policy Guidelines (updated - January 2013). Pretoria: Department of Health; 2013.

2. World Health Organization. WHO treatment guidelines for drug-resistant tuberculosis, 2016 update. WHO/HTM/TB/2016.04. Geneva 2016.

3. South African National Department of Health. Management of Rifampicin-Resistant Tuberculosis. Pretoria, South Africa. November 2019.

4. South African National Department of Health. Clinical management of RR-TB: Updated Clinical Reference Guide. September 2023. Pretoria.

5. World Health Organization. Meeting report of the WHO expert consultation on the definition of extensively drug-resistant tuberculosis, 27-29 October 2020. Geneva: 2021. CC BY-NC-SA 3.0 IGO.
